# Vasopressors and Inotropes in Acute Myocardial Infarction Related Cardiogenic Shock: A Systematic Review and Meta-Analysis

**DOI:** 10.3390/jcm9072051

**Published:** 2020-06-30

**Authors:** Mina Karami, Veemal V. Hemradj, Dagmar M. Ouweneel, Corstiaan A. den Uil, Jacqueline Limpens, Luuk C. Otterspoor, Alexander P. Vlaar, Wim K. Lagrand, José P. S. Henriques

**Affiliations:** 1 Heart Center, Department of Interventional Cardiology, Amsterdam Cardiovascular Sciences, Amsterdam UMC, University of Amsterdam, 1105 AZ Amsterdam, The Netherlands; m.karami@amsterdamumc.nl (M.K.); d.m.ouweneel@amsterdamumc.nl (D.M.O.); 2 Department of Cardiology, Isala, 8025 AB Zwolle, The Netherlands; v.v.hemradj@amsterdamumc.nl; 3 Departments of Cardiology and Intensive Care Medicine, Erasmus MC, University Medical Center, 3015 GD Rotterdam, The Netherlands; c.denuil@erasmusmc.nl; 4 Medical Library, Amsterdam UMC, University of Amsterdam, 1105 AZ Amsterdam, The Netherlands; j.m.limpens@amsterdamumc.nl; 5 Heart Center Catharina Hospital, 5623 EJ Eindhoven, The Netherlands; otterspoor@gmail.com; 6 Department of Intensive Care, Amsterdam UMC, University of Amsterdam, 1105 AZ Amsterdam, The Netherlands; a.p.vlaar@amsterdamumc.nl (A.P.V.); w.k.lagrand@amsterdamumc.nl (W.K.L.)

**Keywords:** cardiogenic shock, inotrope, vasopressor, myocardial infarction, low cardiac output, shock, review

## Abstract

Vasopressors and inotropes are routinely used in acute myocardial infarction (AMI) related cardiogenic shock (CS) to improve hemodynamics. We aimed to investigate the effect of routinely used vasopressor and inotropes on mortality in AMI related CS. A systematic search of MEDLINE, EMBASE and CENTRAL was performed up to 20 February 2019. Randomized and observational studies reporting mortality of AMI related CS patients were included. At least one group should have received the vasopressor/inotrope compared with a control group not exposed to the vasopressor/inotrope. Exclusion criteria were case reports, correspondence and studies including only post-cardiac surgery patients. In total, 19 studies (6 RCTs) were included, comprising 2478 CS patients. The overall quality of evidence was graded low. Treatment with adrenaline, noradrenaline, vasopressin, milrinone, levosimendan, dobutamine or dopamine was not associated with a difference in mortality between therapy and control group. We found a trend toward better outcome with levosimendan, compared with control (RR 0.69, 95% CI 0.47–1.00). In conclusion, we found insufficient evidence that routinely used vasopressors and inotropes are associated with reduced mortality in patients with AMI related CS. Considering the limited evidence, this study emphasizes the need for randomized trials with appropriate endpoints and methodology.

## 1. Introduction

Cardiogenic shock (CS) is a low cardiac output state that leads to hypoperfusion and subsequent multi-organ failure with high mortality rates [1,2]. The commonly used clinical definition of CS is based on hemodynamic criteria and signs of hypoperfusion [3].

Vasopressors and inotropes are routinely used in the treatment of CS to improve hemodynamics and restore organ perfusion. Scientific statements recommend noradrenaline as the first-line pharmacologic treatment strategy in CS, with the addition of inotropes in patients with persistent low cardiac output [4,5].

Acute myocardial infarction (AMI) is a common cause of CS, accounting for at least 30% of the cases [6,7,8]. Vasopressors and inotropes are used in patients with AMI related CS, in the assumption that they improve and maintain adequate coronary perfusion and cardiac output. However, these drugs are also associated with arrhythmias and may increase the myocardial oxygen demand by increasing the contractility, afterload and/or by reducing coronary perfusion [9].

Therefore, we performed a systematic review to examine the current evidence on the effect of different vasopressors and inotropes on mortality in patients with AMI related CS.

In this systematic review and meta-analysis, we addressed the following questions: (1) Is treatment with adrenaline, noradrenaline, vasopressin, milrinone, levosimendan, dobutamine or dopamine associated with reduced mortality in patients with AMI related CS? and (2) What is the effect of these drugs on complications and safety outcomes (e.g., duration of supportive measures, length of intensive care unit (ICU) stay, hemodynamic effects, organ failure and therapy-related complications)?

## 2. Experimental Section

This study followed the Preferred Reporting Items for Systematic Reviews and Meta-Analysis PRISMA guidelines. [10] The protocol was registered in the PROSPERO database (CRD42018107644).

### 2.1. Selection Criteria

To qualify for inclusion, studies must have reported on mortality in CS patients and include patients with AMI. Furthermore, studies were considered for inclusion if outcome data were reported in (A) patients treated with an inotrope or vasopressor and (B) a control group that was not exposed to the (same) inotrope or vasopressor therapy. The following inotropes and vasopressors were included: (1) adrenaline, (2) noradrenaline, (3) vasopressin, (4) milrinone, (5) levosimendan, (6) dobutamine and (7) dopamine. Studies comparing different dosages of inotropes or vasopressors were excluded, unless an unexposed control arm (B) was present. We excluded studies that reported only on post-cardiac surgery patients. We expected very few evidences from randomized controlled trials (RCTs) and therefore we included all study designs except case reports and correspondences.

### 2.2. Search Strategy

A medical information specialist (JL) conducted a systematic search of the following databases: MEDLINE (OVID), EMBASE (OVID), and the Cochrane Central Register of Controlled Trials (CENTRAL) from inception to 20 February 2019. The search included both controlled terms (i.e., MeSH-terms) and free-text words for: (1) cardiogenic shock (or shock /low cardiac output and MI) and (2) inotrope/vasopressor drugs (see Appendix A). Non-human studies, narrative reviews and editorials were excluded. No further restrictions were applied. Reference lists and citing articles of identified relevant papers were crosschecked. The search was adapted in the case of additional relevant studies. The bibliographic records retrieved were imported using EndNote X8 and duplications were removed.

### 2.3. Data Extraction and Quality Assessment

Two researchers (either MK, WL, DO or VH) independently screened titles and abstracts of the retrieved papers and excluded studies based on the selection criteria. Subsequently, two researchers independently assessed the eligibility of the potentially eligible papers in full text. Conference papers were considered for inclusion if they met selection criteria and necessary data were available. After the eligible studies were identified, two researchers (MK and VH) independently performed data extraction. We attempted to retrieve unclear or missing data by contacting the corresponding authors of studies. The quality of the individual studies was assessed using the Revised Cochrane risk-of-bias (RoB 2.0) for RCTs and the Newcastle–Ottawa scale (assessed on mortality outcome level) for non-randomized studies [11,12]. The overall quality of evidence was assessed using the GRADE’s approach [13]. Any discrepancies between the researchers were solved by discussion and/or the involvement of a third researcher (JH).

### 2.4. Data Analysis

The primary outcome was mortality, defined as short-term (<90 day) and long-term (≥90 day) mortality. The therapy group was defined as the examined vasopressor/inotrope. The control group was constructed from the cumulated comparator of the different studies. We summarized mortality in a quantitative manner, using a random-effects model, presented with relative risks (RR) and 95% confidence intervals (CI). Heterogeneity was evaluated using Chi-squared and I-squared test, with the significance set at a *p*-value of 0.10. An I-squared test of >40% was considered indication of substantial heterogeneity. Sensitivity analyses (excluding conference papers and/or observational studies) were applied where appropriate. Review Manager (version 5.3) was used for statistical analysis. We also provided a narrative synthesis of the included studies regarding study population, therapies and outcome(s) and described the following secondary outcomes: duration of supportive measures, length of ICU stay, hemodynamic effects, organ failure and therapy-related complications.

## 3. Results

### 3.1. Search Results

**Figure 1:**
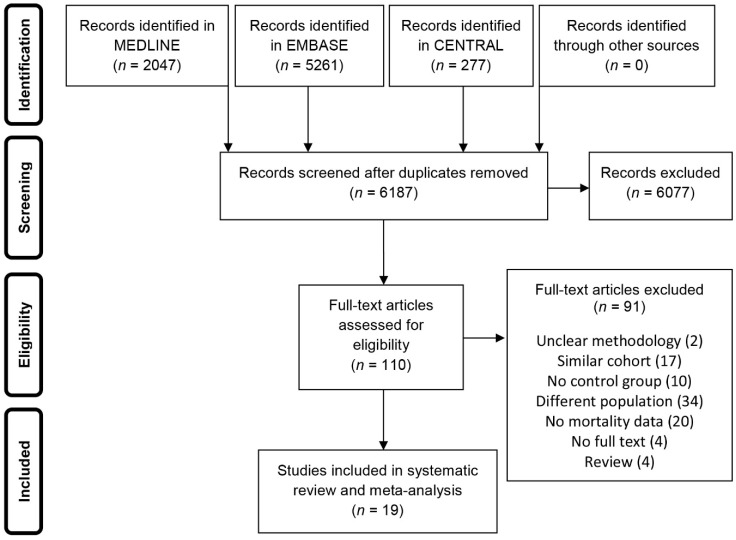
Flowchart of study selection.

Of the 6187 unique publications retrieved, 110 were assessed full text for eligibility and 19 studies were included in the review. The reasons for exclusion are listed in Figure 1.

### 3.2. Study Characteristics

Study characteristics of the 19 included studies are presented in Table 1.

### 3.3. Participants

The overall patient population included in the studies and baseline characteristics of the CS patients are described in the Supplementary Materials, Table S1. In total, 4441 patients were included, of whom 2478 CS patients. All included studies included at least a subgroup with AMI related CS, and 10 studies included only patients with AMI related CS. Overall, there were 137 CS patients treated with adrenaline, 594 CS patients treated with noradrenaline, 8 CS patients treated with vasopressin, 50 CS patients treated with milrinone, 209 CS patients treated with levosimendan, 200 CS patients treated with dobutamine and 367 CS patients treated with dopamine.

### 3.4. Intervention

Criteria for initiation of vasopressor or inotrope therapy differed amongst the various studies and are described in the Supplementary Materials (Table S1).

### 3.5. Comparison

We identified six RCTs with different comparisons [14,15,16,17,18,19]; noradrenaline versus adrenaline in patients with AMI related CS (Levy, 2018), noradrenaline versus adrenaline in patients that required a vasopressor for any cause (Myburgh, 2008), noradrenaline versus dopamine in patients with shock of all-causes (De Backer, 2010 = SOAP II trial), dobutamine versus levosimendan in STEMI patients with CS after PCI (Samimi-Fard, 2007), levosimendan versus placebo in acute STEMI patients with clinical signs of heart failure <48 h after primary PCI (Huseby, 2013 = LEAF trial) and levosimendan versus enoximone in patients with refractory CS < 2 h after PCI (Fuhrmann, 2008). Of note, in all of the included studies, the control group was exposed to some type of vasopressor or inotrope other than the drug that was examined (noradrenaline in most cases).

### 3.6. Quality of Studies

The overall body of evidence regarding the primary outcome was graded low, assessed using the GRADE’s approach. This grade was based on only limited evidence from randomized trials with serious inconsistency and risk of bias. The individual quality assessment of the studies is presented in the Supplementary Materials; Table S2 for the randomized studies and Table S3 for the observational studies.

### 3.7. Mortality Outcomes

**Figure 2:**
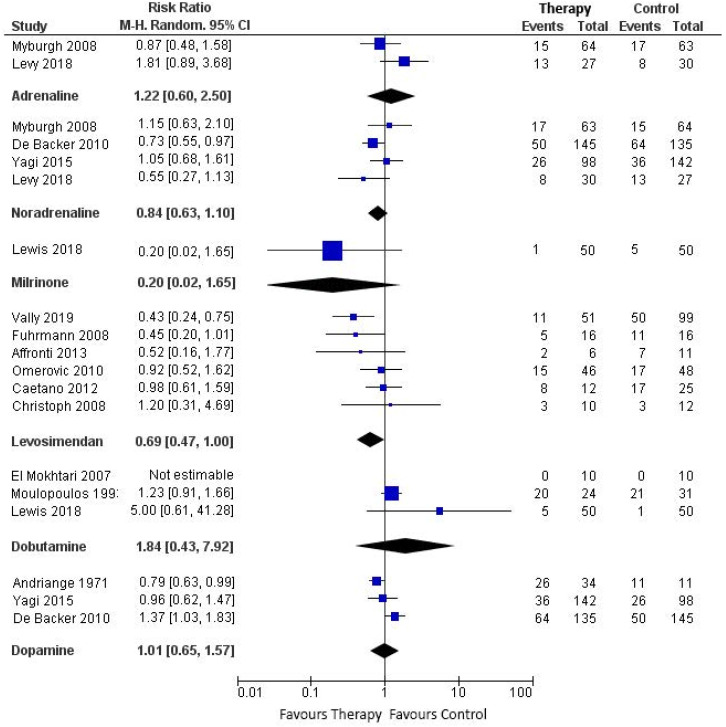
Forest plot demonstrating short-term (<90 day) mortality of cardiogenic shock patients treated with a vasopressor/inotrope versus a constructed control group.

**Figure 3:**
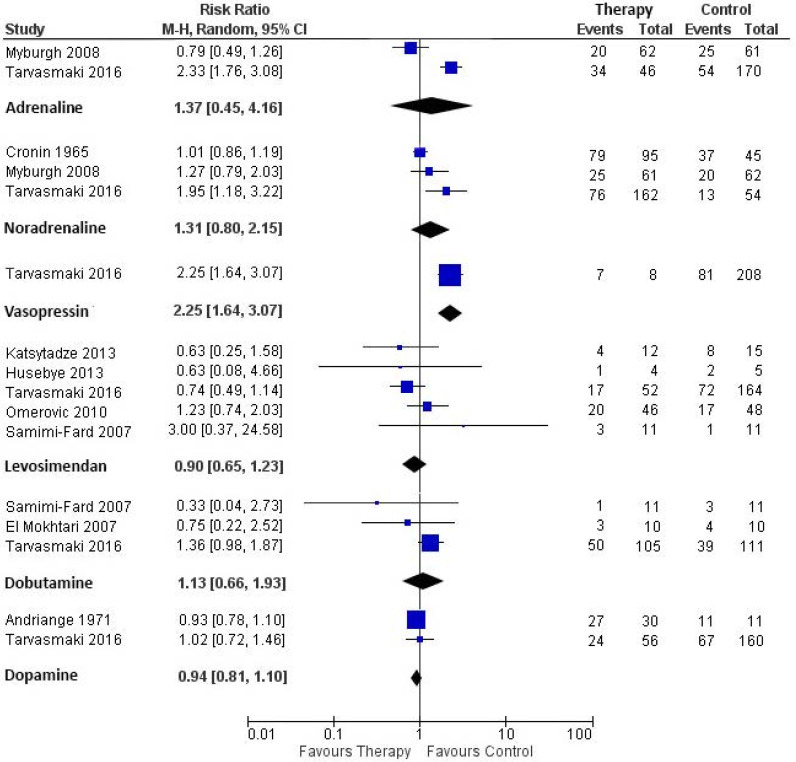
Forest plot demonstrating long-term (≥90 day) mortality of cardiogenic shock patients treated with a vasopressor/inotrope versus a constructed control group.

The synthesis of mortality is shown in Figure 2 for short-term mortality and Figure 3 for long-term mortality. Mortality outcomes of CS patients in the individual studies are described in the Supplementary Materials, Table S4.

#### 3.7.1. Adrenaline vs. Constructed Control

Three of the included studies reported on patients treated with adrenaline [14,15,20]. Levy et al. and Myburgh et al. performed an RCT comparing the effect of adrenaline and noradrenaline on hemodynamic parameters (respectively, cardiac index evolution and achievement of MAP goal > 24 h). Tarvasmaki et al. performed an observational study in which they compared 90-day mortality of patients with acute CS treated with adrenaline (also for noradrenaline, vasopressin, levosimendan, dobutamine, dopamine) versus a non-exposed control group. The two RCTs comparing adrenaline with noradrenaline evaluated 28-day mortality. Their pooled estimate showed no treatment effect of adrenaline on short-term mortality (RR 1.22, 95% CI 0.60–2.50, heterogeneity *I*^2^ = 58%). Pooled estimate of the studies that reported on long-term mortality also showed no treatment effect of adrenaline (RR 1.37 95% CI 0.45–4.16; *n* = 2 studies), with substantial heterogeneity between the studies (*I*^2^ = 94%).

#### 3.7.2. Noradrenaline vs. Constructed Control

Six studies (three RCTs) reported on patients treated with noradrenaline [14,15,16,20,21,22]. Treatment with noradrenaline was not associated with a difference in short-term mortality (RR 0.84, 95% CI 0.63–1.10; *n* = 4 studies, heterogeneity *I*^2^ = 30%). Sensitivity analysis including only the three RCTs that reported on short-term mortality also showed no treatment effect of noradrenaline (RR 0.77, 95% CI 0.56–1.06; *I*^2^ = 26%). Likewise, the pooled estimate showed no treatment effect of noradrenaline on long-term mortality (RR 1.31, 95% CI 0.80-2.15; *n*= 3 studies), with substantial heterogeneity between the studies (*I*^2^ = 81%).

#### 3.7.3. Vasopressin vs. Constructed Control

Mortality outcomes of vasopressin treatment were available in one observational study [20]. In this study 90 day mortality consisted of 7 deaths out of 8 patients (87.5%) treated with vasopressin, compared with 81 deaths out of 208 patients (38.9%) in the control group (RR 2.25, 95% CI 1.64–3.07).

#### 3.7.4. Milrinone vs. Constructed Control

Mortality outcomes of milrinone treatment were available in one observational study [23]. Lewis showed no treatment effect of milrinone on in-hospital mortality, compared with dobutamine. There was 1 death out of 50 patients (2.0%) treated with milrinone, compared with 5 deaths out of 50 patients (10.0%) in the control group (RR 0.20, 95% CI 0.02–1.65).

#### 3.7.5. Levosimendan vs. Constructed Control

Ten studies (three RCTs) reported mortality outcomes of patients treated with levosimendan [17,18,19,20,24,25,26,27,28,29]. In six studies that reported on short-term mortality, the overall trend of the pooled estimate favored levosimendan therapy over the constructed control (RR 0.69, 95% CI 0.47–1.00; participants = 352, heterogeneity *I*^2^ = 39%). In sensitivity analysis excluding the conference paper, levosimendan was associated with favorable outcome, compared to the constructed control (RR 0.61, 95% CI 0.41–0.90, *n* = 5 studies). Pooled estimate showed no treatment effect of levosimendan on long-term mortality (RR 0.90, 95% CI 0.65–1.23; studies, *n* = 5, with low heterogeneity *I*^2^ = 4%). Sensitivity analysis including only the two RCTs that reported long-term mortality showed similar results (RR 0.78, 95% CI 0.36–1.70; *n* = 3 studies). We also performed additional sensitivity analysis for long-term mortality excluding only the conference paper (RR 0.95, 95% CI 0.65–1.40, *n* = 4 studies).

#### 3.7.6. Dobutamine vs. Constructed Control

Five studies (one RCT) reported on patients treated with dobutamine [17,20,23,30,31]. Pooled estimate showed no treatment effect of dobutamine on short-term mortality (RR 1.84, 95% CI 0.43–7.92, substantial heterogeneity *I*^2^ = 56%). Likewise, the pooled estimate of studies that reported on long-term mortality showed no treatment effect of dobutamine (RR1.13, 95% CI 0.66–1.93; *n* = 3 studies, low heterogeneity *I*^2^ = 19%).

#### 3.7.7. Dopamine vs. Constructed Control

Four studies (one RCT) reported on patients treated with dopamine [16,20,22,32]. Three studies (one RCT) reported short-term mortality. The pooled estimate showed no treatment effect of dopamine (RR 1.01 95% CI 0.65–1.57, substantial heterogeneity *I*^2^ = 84%). Sensitivity analysis without the conference paper showed similar results (RR 1.04, 95% CI 0.51–2.12; *n* = 2 studies). There was also no treatment effect of dopamine on long-term mortality (RR 0.94, 95% CI 0.81–1.10; *n*= 2 studies, low heterogeneity *I*^2^ = 0%).

### 3.8. Secondary Outcomes

We summarized the primary endpoint, results and secondary outcomes from the included studies in Table S5, Supplementary Materials. Data on adverse events and safety outcomes were not always available in the studies, but arrhythmic events were reported frequently.

## 4. Discussion

In the present article, we reviewed the current evidence on outcomes of vasopressors and inotropes in patients with AMI related CS and found that adrenaline, noradrenaline, milrinone, levosimendan, dobutamine and dopamine were not associated with a difference in short-term or long-term mortality. The quality of the evidence was low as studies were mostly observational, heterogeneous and included a small number of patients.

We found a positive trend in the pooled estimate of six studies reporting on short-term mortality toward treatment with levosimendan (RR 0.69, 95% CI 0.47–1.00, low-level of evidence). Vasopressin was associated with a difference in long-term mortality in favor of the control group. However, this was based on the results of one observational trial including 8 CS patients treated with vasopressin, compared with a much larger control group. These results are highly biased since the study had a small sample size, was non-randomized and did not adjust for baseline differences.

Our results extend a 2018 Cochrane review with a smaller size (including 13 RCTs; 2001 participants) [33]. The scope of this review was significantly different from ours, since the majority of the included patients had acute on chronic heart failure or cardiac surgery complicated by low cardiac output syndrome (LCOS) or CS, instead of AMI related CS. In this review the effect of inotropes and vasodilators on mortality in patients with LCOS or CS was evaluated, demonstrating low-quality evidence that suggested a short-term mortality benefit of levosimendan compared with dobutamine. None of the other examined inotropes or vasodilators were associated with differences in mortality.

Furthermore, a previous Cochrane review published in 2016, investigated vasopressor therapy for hypotensive shock of all etiologies (mostly septic patients) [34]. Again, in this review patients with AMI related CS were underrepresented and there was no subgroup analysis performed according to shock type. Although none of the vasopressors in this review was associated with a difference in mortality, dopamine significantly increased the risk for arrhythmias compared to noradrenaline.

The ESC 2017 Guidelines for the management of AMI in patients presenting with ST-segment elevation recommend dobutamine as the initial therapy in predominant low cardiac output (Class IIb) [5]. Noradrenaline is recommended in CS and severe hypotension (Class IIb) based on one study (De Backer et al.) reporting a lower rate of arrhythmias and a trend toward lower mortality compared with dopamine in a subgroup analysis [16]. However, there are some methodological concerns regarding this trial since randomization was not stratified and the test for subgroup differences (*p* = 0.87) suggested that the effect was not significant. Also, the subgroup of CS patients was heterogeneous, consisting of AMI, chronic heart failure and post-cardiotomy patients. A recently published RCT examined reduced exposure to noradrenaline through permissive hypotension (lower MAP target) in patients with vasodilatory hypotension aged 65 year or older and found that reduced noradrenaline was not harmful and might even be beneficial [35].

Despite the lack in evidence showing efficacy of treatment with vasopressors and inotropes in AMI related CS, there is a widespread use in clinical practice. Physicians are unlikely to refrain from the use of any vasopressor or inotrope in patients with CS. Of note, none of the included studies in this review had a control group that did not receive any vasopressor or inotrope. Therefore, the current literature doesn’t tell us much about the effectiveness of any agent vs. placebo but only against each other. In other words, these drugs are either equally effective or equally ineffective. Furthermore, it is important to realize that hemodynamic parameters do not necessarily correlate with tissue perfusion and patient outcomes. It has not been proven that hemodynamic improvement in CS patients results in better outcome. Currently, CS is a clinical diagnosis that has not been well defined, and there is significant heterogeneity in CS definition among the studies used for meta-analysis. Therefore, the current level of evidence is insufficient to draw conclusions on whether vasopressors/inotropes are effective. However, we do believe it is of clinical importance to review and summarize the current evidence regarding routinely used therapies. This systematic review and meta-analysis is the first to report outcomes of commonly used vasopressor/inotropic therapies in patients with CS complicating AMI.

The authors believe that rigorous studies exploring the efficacy of vasopressors and inotropes with appropriate endpoint and methodology are advocated. Not only to compare the efficacy between different drugs and dosages but also to ascertain whether these drugs are effective in reducing mortality. Several RCTs that challenged the current pharmacological treatment in patients with out-of-hospital cardiac arrest (OHCA) demonstrated that although the administration of adrenaline was associated with improved short-term survival in OHCA, it did not affect survival with favorable neurological outcome [36,37,38]. It is important to also initiate large placebo-controlled, double-blind, RCTs in CS patients to definitively establish the effect of pharmacological therapy. Studying the CS population in an RCT is challenging but feasible with the adoption of adequate methodology and statistical plan analysis.

In the present study, several decisions were made by the research team that could possibly limit our conclusion. The aim of this study was to summarize the evidence regarding vasopressor and inotropic treatment of a selected patient group (AMI related CS), since patients presenting with a non-hypotensive low-output status differ significantly in their treatment goal and strategy. Due to the limited number of studies exclusively reporting on patients with AMI related CS, we also included studies with mixed etiologies, as long as there were AMI patients included in the cohort. 

Another limitation of our review was that the included studies were very heterogeneous in terms of participants, interventions, comparisons and outcomes. Specific subgroup analysis to reduce heterogeneity was not possible, due to the limited number of studies that were available regarding the different drugs. Moreover, single center, retrospective studies with a small sample size were included, which are likely to have significant selection bias. Furthermore, we included all study designs, without any publication or language restriction. It may be debatable to include conference papers (of which the methodological quality could not be properly assessed due to limited information). However, including conference papers may also reduce the effect of potential publication bias. Moreover, randomized evidence is very limited, and observational data is informative in assessing therapy-related complications and safety outcomes. To examine the best possible level of evidence, we performed sensitivity analyses where possible excluding the conference papers and/or observational studies.

## 5. Conclusions

There is insufficient evidence that routinely used vasopressors and inotropes are associated with reduced mortality in patients with AMI related CS. Considering the limited evidence, this study emphasizes the need for proper randomized trials.

## Figures and Tables

**Table 1:** Summary of included studies (*n* = 19) on inotrope/vasopressor therapy in cardiogenic shock.

Study	Year	Country	Center	Setting	Inclusion Period	Follow-up	Overall N	CS N
Cronin	1965	Canada	Single	Retrospective cohort	1952–1961	10 years	140	140
Moulopoulos	1993	Greece	Single	Retrospective cohort	1978–1991	1 month	55	55
Andriange	1971	Belgium	Single	Retrospective cohort	1967–1970	1 year	450	45
Samimi-Fard	2007	Spain	Single	Randomized trial	2003–2004	1 year	22	22
El Mokhtari	2007	Germany	Single	Retrospective cohort	-	1 year	20	20
Fuhrmann	2008	Germany	Single	Randomized trial	2003–2005	30 days	32	32
Myburgh	2008	Australia	Multi	Randomized trial	2004–2006	90 days	280	128
Christoph	2008	Germany	Single	Prospective cohort	2003–2005	-	22	22
De Backer	2010	Belgium	Multi	Randomized trial	2003–2007	1 year	1679	280
Omerovic	2010	Sweden	Single	Prospective cohort	2004–2006	1 year	94	94
Caetano	2012	Portugal	-	Retrospective cohort (conference paper)	-	10.6 ± 10.9 months	37	37
Huseby	2013	Norway	Single	Randomized trial	2006–2010	6 months	61	9
Affronti	2013	Italy	Single	Retrospective case-control	2011	-	17	17
Katsytadze	2013	Ukraine	-	Retrospective cohort (conference paper)	-	1 year	27	27
Yagi	2015	Japan	Multi	Prospective cohort (conference paper)	2012–2014	30 days	979	240
Tarvasmaki	2016	Finland	Multi	Prospective cohort	2010–2012	90 days	216	216
Levy	2018	France	Multi	Randomized trial	2011–2016	60 days	57	57
Vally	2019	France	Single	Retrospective cohort	2010–2017	30 days	150	150
Lewis	2018	USA	Single	Retrospective cohort	2013–2015	In-hospital	100	100

CS: cardiogenic shock.

## Supplementary Materials

The following are available online at https://www.mdpi.com/2077-0383/9/7/2051/s1. Table S1: Overall patient population and baseline characteristics. Table S2: Quality assessment of the randomized trials. Table S3: Quality assessment of the observational studies. Table S4: Mortality outcomes for cardiogenic shock population. Table S5: Primary and secondary outcome(s) of the included studies.

## Appendix A. Search strategies

Medline

Database(s): Ovid MEDLINE(R) and Epub Ahead of Print, In-Process & Other Non-Indexed Citations and Daily 1946 to February 19, 2019

Search Strategy: 2019-02-20

**#**	**Searches**	**Results**
1	shock, cardiogenic/	7757
2	(cardiogen* adj9 shock*).tw,ot,kw,kf.	10393
3	(circulator* adj3 shock*).tw,ot,kf.	1251
4	coron* shock*.tw,ot,kw,kf.	20
5	acute circulatory fail*.tw,ot,kw.	247
6	*shock/dt not (septic or sepsis or bacter*em* or infect* or endotoxin* or heat shock or HSP* or anaphyl* or allergic or vasodilat* or osmotic or h?emorr* or bleeding or toxic or neurogen* or burn* or shock-wave or shock-resistant or hypovolemic or electroconvuls* or distributive shock*).ti.	711
7	((undifferent* or undiagnos* or different or various or variety or type or types or kind or kinds or forms or states or etiologies) adj2 (shock* not heat shock*)).tw.	1662
**8**	**or/1-7 [I = cardiogenic shock ]**	**16778**
9	myocardial ischemia/ or acute coronary syndrome/ or exp myocardial infarction/ or percutaneous coronary intervention/ or angioplasty, balloon, coronary/ or myocardial revascularization/ or coronary artery bypass/	275348
10	(((myocard* or heart or card* or ST) adj3 (infarct* or isch?em*)) or (coronary intervent* or STEMI or PCI or angioplast* or post-isch* or postisch* or postinfarct* or post-infarct* or MI or AMI or myocard* revasc* or coronary artery bypass* or CABG or aortocoronary bypass* or coronary bypass*)).tw,ot,kf.	379295
**11**	**9 or 10 [MI]**	**454472**
12	(shock/ or shock.ti,kw.) not (electric countershock/ or (septic or sepsis or bacter*em* or infect* or endotoxin* or heat shock or HSP* or anaphyl* or allergic or vasodilat* or osmotic or h?emorr* or bleeding or toxic or neurogen* or burn* or shock-wave or shock-resistant or hypovolemic or electroconvuls* or distributive shock*).ti.) [shock general]	30283
13	cardiac output, low/	5447
14	((low* or diminish* or decreas* or declin* or reduc* or negligibl* or fall*) adj2 (card* or heart) adj output*).tw,kf.	8066
15	((low* or diminish* or decreas* or declin* or reduc* or negligibl*) adj output*).tw,kf.	1988
16	cardiac output low.tw,kf,ot.	51
17	(LCOS or COS or LCO).tw,ot,kw.	17758
18	((instab* or unstab*) adj1 h?emodyn*).tw,kf.	7625
**19**	**or/12-18 [shock, low CO]**	**69271**
**20**	**11 and 19 [ II = MI + CO ]**	**8371**
**21**	**8 or 20 [ I II ]**	**20946**
22	(animals/ not humans/) or (porcine or piglet or pig or pigs or rat or rats or dogs or dog or canine).ti. or ph?eochrom*.mp.	4677715
23	(review or editorial).pt. not ((case reports or clinical trial or clinical trial phase iii or clinical trial phase iv or comparative study or controlled clinical trial or evaluation studies or letter or meta analysis or randomized controlled trial).pt. or case-control studies/ or exp cohort studies/ or cross-sectional studies/ or (systematic adj3 (literature or review)).tw,ot,kf.)	2590121
**24**	**22 or 23**	**7115287**
**25**	**21 not 24 [ I II - human not review,editorial]**	**16394**
26	cardiotonic agents/tu, ad	8559
27	catecholamines/ad, tu	948
28	(cardiotonic* or inopressor* or inodilator*).tw,ot,kw.	2445
29	(catecholamines or inotropes or vasopressors or ((catecholamine or inotrop* or vasopressor) adj3 (support or solution* or infusion* or dosing or dose* or dosage* or prescri* or "use" or therap* or treat* or cotreat* or administ* or requir* or need* or superior or first-line or "add" or added or addition or adding or receiv* or receipt or regimen* or alone or combin* or plus or compar* or vs or versus or assigned or allocat* or randomi?ed or effectiveness or efficacy or drug* or agent* or substance* or group))).tw,ot,kf.	48357
30	dopamine/	67481
31	(dopamin* or hydroxytyramin* or Intropin*).tw,ot,kw.	145939
32	dobutamine/	6002
33	dobutamin*.tw,ot,kw.	8454
34	noradrenaline/	84076
35	(norepinephrin* or noradrenalin*).tw,ot,kw.	92575
36	adrenaline/	54084
37	(epinephrin* or adrenalin*).tw,ot,kw.	56754
38	milrinone/	1316
39	milrinon*.tw,ot,kw.	1847
40	vasopressins/	21322
41	vasopressin*.tw,ot,kw.	33741
42	simendan/	1042
43	(simendan or levosimend*).tw,ot,kw.	1286
**44**	**or/26-43 [ inotropes/vasopressors ]**	**378001**
45	25 and 44 [ cardiogenic shock + inotropes/vasopressors ]	2050
**46**	**remove duplicates from 45 [ cardiogenic shock + inotropes/vasopressors -deduplicated ]**	**2047**

Embase

Database(s): Embase Classic+Embase 1947 to 2019 February 19

Search Strategy: 2019-02-20

**#**	**Searches**	**Results**
1	cardiogenic shock/	24059
2	(cardiogen* adj9 shock*).tw,ot,kw.	18652
3	(circulator* adj3 shock*).tw,ot,kw.	1949
4	coron* shock*.tw,ot,kw.	32
5	acute circulatory fail*.tw,ot,kw.	465
6	*Shock/dt not (septic or sepsis or bacter*em* or infect* or endotoxin* or heat shock or HSP* or anaphyl* or allergic or vasodilat* or osmotic or h?emorr* or bleeding or toxic or neurogen* or burn* or shock-wave or shock-resistant or hypovolemic or electroconvuls* or distributive shock*).ti.	1157
7	((undifferent* or undiagnos* or different or various or variety or type or types or kind or kinds or forms or states or etiologies) adj2 (shock* not heat shock*)).tw.	2735
**8**	**or/1-7 [ I ]**	**32877**
9	heart muscle ischemia/ or heart infarction/ or acute heart infarction/ or anterior myocardial infarction/ or heart infarction size/ or exp heart ventricle infarction/ or non st segment elevation myocardial infarction/ or posterior myocardial infarction/ or st segment elevation myocardial infarction/ or exp percutaneous coronary intervention/ or percutaneous transluminal angioplasty/ or coronary artery bypass graft/ or coronary artery bypass surgery/	547773
10	(((myocard* or heart or card* or ST) adj3 (infarct* or isch?em*)) or (coronary intervent* or STEMI or PCI or angioplast* or post-isch* or postisch* or postinfarct* or post-infarct* or MI or AMI or myocard* revasc* or coronary artery bypass* or CABG or aortocoronary bypass* or coronary bypass*)).tw,ot,kw.	572593
**11**	**9 or 10 [MI]**	**737611**
12	(*shock/ or shock.ti.) not (exp heart stimulation/ or (septic or sepsis or bacter*em* or infect* or endotoxin* or heat shock or HSP* or anaphyl* or allergic or vasodilat* or osmotic or h?emorr* or bleeding or toxic or neurogen* or burn* or shock-wave or shock-resistant or hypovolemic or electroconvuls* or distributive shock*).ti.) [shock general]	32315
13	forward heart failure/	5518
14	((low* or diminish* or decreas* or declin* or reduc* or negligibl* or fall*) adj2 (card* or heart) adj output*).tw,kw.	12569
15	((low* or diminish* or decreas* or declin* or reduc* or negligibl*) adj output*).tw,kw.	2964
16	cardiac output low.tw,kw,ot.	105
17	(LCOS or COS or LCO).tw,ot,kw.	20242
18	((instab* or unstab*) adj1 h?emodyn*).tw,kw.	12677
**19**	**or/12-18 [shock, low CO]**	**81903**
**20**	**11 and 19 [ II = MI + CO ]**	**10001**
**21**	**8 or 20 [I II]**	**38501**
22	(book or editorial).pt. or book/ or editorial/ or ((("review" or letter).pt. or "review"/ or letter/) not (meta analysis/ or "systematic review"/ or clinical study/ or case control study/ or exp controlled clinical trial/ or controlled study/ or intervention study/ or longitudinal study/ or major clinical study/ or prospective study/ or retrospective study/ or cohort analysis/ or (systematic adj2 (literature or review)).tw,ot,kw.))	3896039
23	((animal/ or animal experiment/ or animal model/ or nonhuman/) not human/) or (porcine or piglet or pig or pigs or rat or rats or dogs or dog or canine).ti. or ph?eochrom*.mp.	6420601
**24**	**22 or 23**	**10059750**
**25**	**21 not 24 [I II - humans not editorials, reviews]**	**30861**
26	*inotropic agent/ or inotropic agent/ae, ct, cb, cm, dt	6390
27	*cardiotonic agent/ or cardiotonic agent/ae, ct, cb, cm, dt	2726
28	catecholamine/ae, ct, cb, cm, dt	1671
29	(cardiotonic* or inopressor* or inodilator*).tw,ot,kw.	3739
30	(catecholamines or inotropes or vasopressors or ((catecholamine or inotrop* or vasopressor) adj3 (support or solution* or infusion* or dosing or dose* or dosage* or prescri* or "use" or therap* or treat* or cotreat* or administ* or requir* or need* or superior or first-line or "add" or added or addition or adding or receiv* or receipt or regimen* or alone or combin* or plus or compar* or vs or versus or assigned or allocat* or randomi?ed or effectiveness or efficacy or drug* or agent* or substance* or group))).tw,ot,kw.	74053
31	dopamine/ not (dopamine/ec not dopamine/ae, cb, cm, dt)	80557
32	(dopamin* or hydroxytyramin* or Intropin*).tw,ot,kw.	189228
33	dobutamine/ not (dobutamine/ec not dobutamine/ae, cb, cm, dt)	23435
34	dobutamin*.tw,ot,kw.	12411
35	noradrenalin/ not (noradrenalin/ec not noradrenalin/ae, cb, cm, dt)	114540
36	(norepinephrin* or noradrenalin*).tw,ot,kw.	120459
37	adrenalin/ not (adrenalin/ec not adrenalin/ae, cb, cm, dt)	111164
38	(epinephrin* or adrenalin*).tw,ot,kw.	83979
39	milrinone/	7278
40	milrinon*.tw,ot,kw.	2706
41	vasopressin/ not (vasopressin/ec not vasopressin/ad, cb, cm, dt)	29150
42	vasopressin*.tw,ot,kw.	41607
43	simendan/	106
44	(simendan or levosimend*).tw,ot,kw.	2165
**45**	**or/26-44 [drugs]**	**549646**
**46**	**25 and 45 [total]**	**5721**
**47**	**remove duplicates from 46 [total hits -deduplicated]**	**5640**
48	(embase or elsevier or canadian).cr.	26116001
**49**	**47 and 48 [ EMBASE records only]**	**5261**

**CENTRAL**

**ID**	**Search**	**Hits**
#1	(cardiogen* near/9 shock*):ti,ab,kw	914
#2	(circulator* near/3 shock*):ti,ab,kw	36
#3	(coron* NEXT shock*):ti,ab,kw	0
#4	(acute NEXT circulatory NEXT fail*):ti,ab,kw	14
#5	((undifferent* or undiagnos* or different or various or variety or type or types or kind or kinds or forms or states or etiologies) near/2 shock*):ti,ab not (heat NEXT shock*):ti,ab	82
**#6**	**#1 or #2 or #3 or #4 or #5**	**1036**
**#7**	**(((myocard* or heart or card* or ST) near/3 (infarct* or isch*em*)) or ((coronary NEXT intervent*) or STEMI or PCI or angioplast* or (post NEXT isch*) or postisch* or postinfarct* or (post NEXT infarct*) or MI or AMI or (myocard* NEXT revasc*) or (coronary NEXT artery NEXT bypass*) or CABG or (aortocoronary NEXT bypass*) or (coronary NEXT bypass*)))**	**57969**
#8	shock:ti,kw not (septic or sepsis or bacter*em* or infect* or endotoxin* or (heat NEXT shock) or HSP* or anaphyl* or allergic or vasodilat* or osmotic or h*emorr* or bleeding or toxic or neurogen* or burn* or (shock NEXT wave) or (shock NEXT resistant) or hypovolemic or (hypo NEXT volemic) or electroconvuls* or (distributive NEXT shock*)):ti	2559
#9	((low* or diminish* or decreas* or declin* or reduc* or negligibl* or fall*) near/2 ((card* NEXT output) OR (heart NEXT output))):ti,ab,kw	1094
#10	((low* or diminish* or decreas* or declin* or reduc* or negligibl*) next output*):ti,ab,kw	138
#11	(LCOS or COS or LCO):ti,ab,kw	312
#12	((instab* or unstab*) near/1 h*emodyn*):ti,ab,kw	633
**#13**	**#8 or #9 or #10 #11 or #12**	**4238**
**#14**	**#7 and #13**	**1078**
**#15**	**#6 or #14**	**1595**
#16	(cardiotonic* or inopressor* or inodilator* or dopamin* or hydroxytyramin* or Intropin* or dobutamin* or norepinephrin* or noradrenalin* or epinephrin* or adrenalin* or milrinon* or vasopressin* or simendan or levosimend*):ti,ab,kw	22220
#17	(catecholamines or inotropes or vasopressors or ((catecholamine or inotrop* or vasopressor) near/3 (support or solution* or infusion* or dosing or dose* or dosage* or prescri* or "use" or therap* or treat* or cotreat* or administ* or requir* or need* or superior or (first NEXT line) or "add" or added or addition or adding or receiv* or receipt or regimen* or alone or combin* or plus or compar* or vs or versus or assigned or allocat* or randomi*ed or effectiveness or efficacy or drug* or agent* or substance* or group))):ti,ab,kw	4646
**#18**	**#16 or #17**	**24628**
**#19**	**#15 and #18**	**277**
