# Supplementary material for: Vasopressors and Inotropes in Acute Myocardial Infarction Related Cardiogenic Shock: A Systematic Review and Meta-Analysis
Source: J Clin Med. 2020 Jun 30;9(7):2051. doi: 10.3390/jcm9072051 (PMC7408805; doi:10.3390/jcm9072051)
Supplement: Supplementary file 1 [file jcm-09-02051-s001.pdf]

APPENDIX

Appendix A. Search strategies.

MEDLINE

Database(s): **Ovid MEDLINE(R) and Epub Ahead of Print, In-Process & Other Non-Indexed Citations and Daily** 1946 to February 19, 2019
Search Strategy: **2019-02-20**

#	Searches	Results
1	shock, cardiogenic/	7757
2	(cardiogen* adj9 shock*).tw,ot,kw,kf.	10393
3	(circulator* adj3 shock*).tw,ot,kf.	1251
4	coron* shock*.tw,ot,kw,kf.	20
5	acute circulatory fail*.tw,ot,kw.	247
6	*shock/dt not (septic or sepsis or bacter*em* or infect* or endotoxin* or heat shock or HSP* or anaphyl* or allergic or vasodilat* or osmotic or h?emorr* or bleeding or toxic or neurogen* or burn* or shock-wave or shock-resistant or hypovolemic or electroconvuls* or distributive shock*).ti.	711
7	((undifferent* or undiagnos* or different or various or variety or type or types or kind or kinds or forms or states or etiologies) adj2 (shock* not heat shock*)).tw.	1662
**8**	**or/1-7 [I = cardiogenic shock]**	**16778**
9	myocardial ischemia/ or acute coronary syndrome/ or exp myocardial infarction/ or percutaneous coronary intervention/ or angioplasty, balloon, coronary/ or myocardial revascularization/ or coronary artery bypass/	275348
10	(((myocard* or heart or card* or ST) adj3 (infarct* or isch?em*)) or (coronary intervent* or STEMI or PCI or angioplast* or post-isch* or postisch* or postinfarct* or post-infarct* or MI or AMI or myocard* revasc* or coronary artery bypass* or CABG or aortocoronary bypass* or coronary bypass*)).tw,ot,kf.	379295
**11**	**9 or 10 [MI]**	**454472**
12	(shock/ or shock.ti,kw.) not (electric countershock/ or (septic or sepsis or bacter*em* or infect* or endotoxin* or heat shock or HSP* or anaphyl* or allergic or vasodilat* or osmotic or h?emorr* or bleeding or toxic or neurogen* or burn* or shock-wave or shock-resistant or hypovolemic or electroconvuls* or distributive shock*).ti.) [shock general]	30283
13	cardiac output, low/	5447
14	((low* or diminish* or decreas* or declin* or reduc* or negligibl* or fall*) adj2 (card* or heart) adj output*).tw,kf.	8066
15	((low* or diminish* or decreas* or declin* or reduc* or negligibl*) adj output*).tw,kf.	1988
16	cardiac output low.tw,kf,ot.	51
17	(LCOS or COS or LCO).tw,ot,kw.	17758
18	((instab* or unstab*) adj1 h?emodyn*).tw,kf.	7625
**19**	**or/12-18 [shock, low CO]**	**69271**
**20**	**11 and 19 [II = MI + CO]**	**8371**
**21**	**8 or 20 [I II]**	**20946**
22	(animals/ not humans/) or (porcine or piglet or pig or pigs or rat or rats or dogs or dog or canine).ti. or ph?eochrom*.mp.	4677715

23	(review or editorial).pt. not ((case reports or clinical trial or clinical trial phase iii or clinical trial phase iv or comparative study or controlled clinical trial or evaluation studies or letter or meta analysis or randomized controlled trial).pt. or case-control studies/ or exp cohort studies/ or cross-sectional studies/ or (systematic adj3 (literature or review)).tw,ot,kf.)	2590121
**24**	**22 or 23**	**7115287**
**25**	**21 not 24 [I II - human not review,editorial]**	**16394**
26	cardiotonic agents/tu, ad	8559
27	catecholamines/ad, tu	948
28	(cardiotonic* or inopressor* or inodilator*).tw,ot,kw.	2445
29	(catecholamines or inotropes or vasopressors or ((catecholamine or inotrop* or vasopressor) adj3 (support or solution* or infusion* or dosing or dose* or dosage* or prescri* or "use" or therap* or treat* or cotreat* or administ* or requir* or need* or superior or first-line or "add" or added or addition or adding or receiv* or receipt or regimen* or alone or combin* or plus or compar* or vs or versus or assigned or allocat* or randomi?ed or effectiveness or efficacy or drug* or agent* or substance* or group))).tw,ot,kf.	48357
30	dopamine/	67481
31	(dopamin* or hydroxytyramin* or Intropin*).tw,ot,kw.	145939
32	dobutamine/	6002
33	dobutamin*.tw,ot,kw.	8454
34	noradrenaline/	84076
35	(norepinephrin* or noradrenalin*).tw,ot,kw.	92575
36	adrenaline/	54084
37	(epinephrin* or adrenalin*).tw,ot,kw.	56754
38	milrinone/	1316
39	milrinon*.tw,ot,kw.	1847
40	vasopressins/	21322
41	vasopressin*.tw,ot,kw.	33741
42	simendan/	1042
43	(simendan or levosimend*).tw,ot,kw.	1286
**44**	**or/26-43 [inotropes/vasopressors]**	**378001**
45	25 and 44 [cardiogenic shock + inotropes/vasopressors]	2050
**46**	**remove duplicates from 45 [cardiogenic shock + inotropes/vasopressors - deduplicated]**	**2047**

Embase

Database(s): **Embase Classic+Embase** 1947 to 2019 February 19
Search Strategy: **2019-02-20**

#	Searches	Results
1	cardiogenic shock/	24059
2	(cardiogen* adj9 shock*).tw,ot,kw.	18652
3	(circulator* adj3 shock*).tw,ot,kw.	1949
4	coron* shock*.tw,ot,kw.	32
5	acute circulatory fail*.tw,ot,kw.	465

6	*Shock/dt not (septic or sepsis or bacter*em* or infect* or endotoxin* or heat shock or HSP* or anaphyl* or allergic or vasodilat* or osmotic or h?emorr* or bleeding or toxic or neurogen* or burn* or shock-wave or shock-resistant or hypovolemic or electroconvuls* or distributive shock*).ti.	1157
7	((undifferent* or undiagnos* or different or various or variety or type or types or kind or kinds or forms or states or etiologies) adj2 (shock* not heat shock*)).tw.	2735
**8**	**or/1-7 [I]**	**32877**
9	heart muscle ischemia/ or heart infarction/ or acute heart infarction/ or anterior myocardial infarction/ or heart infarction size/ or exp heart ventricle infarction/ or non st segment elevation myocardial infarction/ or posterior myocardial infarction/ or st segment elevation myocardial infarction/ or exp percutaneous coronary intervention/ or percutaneous transluminal angioplasty/ or coronary artery bypass graft/ or coronary artery bypass surgery/	547773
10	(((myocard* or heart or card* or ST) adj3 (infarct* or isch?em*)) or (coronary intervent* or STEMI or PCI or angioplast* or post-isch* or postisch* or postinfarct* or post-infarct* or MI or AMI or myocard* revasc* or coronary artery bypass* or CABG or aortocoronary bypass* or coronary bypass*)).tw,ot,kw.	572593
**11**	**9 or 10 [MI]**	**737611**
12	(*shock/ or shock.ti.) not (exp heart stimulation/ or (septic or sepsis or bacter*em* or infect* or endotoxin* or heat shock or HSP* or anaphyl* or allergic or vasodilat* or osmotic or h?emorr* or bleeding or toxic or neurogen* or burn* or shock-wave or shock-resistant or hypovolemic or electroconvuls* or distributive shock*).ti.) [shock general]	32315
13	forward heart failure/	5518
14	((low* or diminish* or decreas* or declin* or reduc* or negligibl* or fall*) adj2 (card* or heart) adj output*).tw,kw.	12569
15	((low* or diminish* or decreas* or declin* or reduc* or negligibl*) adj output*).tw,kw.	2964
16	cardiac output low.tw,kw,ot.	105
17	(LCOS or COS or LCO).tw,ot,kw.	20242
18	((instab* or unstab*) adj1 h?emodyn*).tw,kw.	12677
**19**	**or/12-18 [shock, low CO]**	**81903**
**20**	**11 and 19 [II = MI + CO]**	**10001**
**21**	**8 or 20 [I II]**	**38501**
22	(book or editorial).pt. or book/ or editorial/ or ((("review" or letter).pt. or "review"/ or letter/) not (meta analysis/ or "systematic review"/ or clinical study/ or case control study/ or exp controlled clinical trial/ or controlled study/ or intervention study/ or longitudinal study/ or major clinical study/ or prospective study/ or retrospective study/ or cohort analysis/ or (systematic adj2 (literature or review)).tw,ot,kw.))	3896039
23	((animal/ or animal experiment/ or animal model/ or nonhuman/) not human/) or (porcine or piglet or pig or pigs or rat or rats or dogs or dog or canine).ti. or ph?eochrom*.mp.	6420601
**24**	**22 or 23**	**10059750**
**25**	**21 not 24 [I II - humans not editorials, reviews]**	**30861**
26	*inotropic agent/ or inotropic agent/ae, ct, cb, cm, dt	6390
27	*cardiotonic agent/ or cardiotonic agent/ae, ct, cb, cm, dt	2726
28	catecholamine/ae, ct, cb, cm, dt	1671
29	(cardiotonic* or inopressor* or inodilator*).tw,ot,kw.	3739

30	(catecholamines or inotropes or vasopressors or ((catecholamine or inotrop* or vasopressor) adj3 (support or solution* or infusion* or dosing or dose* or dosage* or prescri* or "use" or therap* or treat* or cotreat* or administ* or requir* or need* or superior or first-line or "add" or added or addition or adding or receiv* or receipt or regimen* or alone or combin* or plus or compar* or vs or versus or assigned or allocat* or randomi?ed or effectiveness or efficacy or drug* or agent* or substance* or group))).tw,ot,kw.	74053
31	dopamine/ not (dopamine/ec not dopamine/ae, cb, cm, dt)	80557
32	(dopamin* or hydroxytyramin* or Intropin*).tw,ot,kw.	189228
33	dobutamine/ not (dobutamine/ec not dobutamine/ae, cb, cm, dt)	23435
34	dobutamin*.tw,ot,kw.	12411
35	noradrenalin/ not (noradrenalin/ec not noradrenalin/ae, cb, cm, dt)	114540
36	(norepinephrin* or noradrenalin*).tw,ot,kw.	120459
37	adrenalin/ not (adrenalin/ec not adrenalin/ae, cb, cm, dt)	111164
38	(epinephrin* or adrenalin*).tw,ot,kw.	83979
39	milrinone/	7278
40	milrinon*.tw,ot,kw.	2706
41	vasopressin/ not (vasopressin/ec not vasopressin/ad, cb, cm, dt)	29150
42	vasopressin*.tw,ot,kw.	41607
43	simendan/	106
44	(simendan or levosimend*).tw,ot,kw.	2165
**45**	**or/26-44 [drugs]**	**549646**
**46**	**25 and 45 [total]**	**5721**
**47**	**remove duplicates from 46 [total hits -deduplicated]**	**5640**
48	(embase or elsevier or canadian).cr.	26116001
**49**	**47 and 48 [EMBASE records only]**	**5261**

CENTRAL

ID	Search	Hits
#1	(cardiogen* near/9 shock*):ti,ab,kw	914
#2	(circulator* near/3 shock*):ti,ab,kw	36
#3	(coron* NEXT shock*):ti,ab,kw	0
#4	(acute NEXT circulatory NEXT fail*):ti,ab,kw	14
#5	((undifferent* or undiagnos* or different or various or variety or type or types or kind or kinds or forms or states or etiologies) near/2 shock*):ti,ab not (heat NEXT shock*):ti,ab	82
**#6**	**#1 or #2 or #3 or #4 or #5**	**1036**
#7	**(((myocard* or heart or card* or ST) near/3 (infarct* or isch*em*)) or ((coronary NEXT intervent*) or STEMI or PCI or angioplast* or (post NEXT isch*) or postisch* or postinfarct* or (post NEXT infarct*) or MI or AMI or (myocard* NEXT revasc*) or (coronary NEXT artery NEXT bypass*) or CABG or (aortocoronary NEXT bypass*) or (coronary NEXT bypass*)))**	**57969**
#8	shock:ti,kw not (septic or sepsis or bacter*em* or infect* or endotoxin* or (heat NEXT shock) or HSP* or anaphyl* or allergic or vasodilat* or osmotic or h*emorr* or bleeding or toxic or neurogen* or burn* or (shock NEXT wave) or (shock NEXT resistant) or hypovolemic or (hypo NEXT volemic) or electroconvuls* or (distributive NEXT shock*)):ti	2559

#9	((low* or diminish* or decreas* or declin* or reduc* or negligibl* or fall*) near/2 ((card* NEXT output) OR (heart NEXT output))):ti,ab,kw	1094
#10	((low* or diminish* or decreas* or declin* or reduc* or negligibl*) next output*):ti,ab,kw	138
#11	(LCOS or COS or LCO):ti,ab,kw	312
#12	((instab* or unstab*) near/1 h*emodyn*):ti,ab,kw	633
**#13**	**#8 or #9 or #10 #11 or #12**	**4238**
**#14**	**#7 and #13**	**1078**
**#15**	**#6 or #14**	**1595**
#16	(cardiotonic* or inopressor* or inodilator* or dopamin* or hydroxytyramin* or Intropin* or dobutamin* or norepinephrin* or noradrenalin* or epinephrin* or adrenalin* or milrinon* or vasopressin* or simendan or levosimend*):ti,ab,kw	22220
#17	(catecholamines or inotropes or vasopressors or ((catecholamine or inotrop* or vasopressor) near/3 (support or solution* or infusion* or dosing or dose* or dosage* or prescri* or "use" or therap* or treat* or cotreat* or administ* or requir* or need* or superior or (first NEXT line) or "add" or added or addition or adding or receiv* or receipt or regimen* or alone or combin* or plus or compar* or vs or versus or assigned or allocat* or randomi*ed or effectiveness or efficacy or drug* or agent* or substance* or group))):ti,ab,kw	4646
**#18**	**#16 or #17**	**24628**
**#19**	**#15 and #18**	**277**

SUPPLEMENTARY MATERIALS

Table S1. Overall patient population and baseline characteristics of cardiogenic shock patients in the included studies.

Study	Overall patient population	Criteria for therapy initiation	Control arm	CS patients (n)		Age (years)		Male (%)		AMI (%)		Revascularization (%)	
				Therapy	Control	Therapy	Control	Therapy	Control	Therapy	Control	Therapy	Control
**Adrenaline**													
Myburgh	Requirement of vasopressor for any cause		Noradrenaline	64	64								
Tarvasmaki	Acute CS	CS	Non exposed	46	170	68±9	66±12	72	75	87	79		
Levy	CS after AMI	CS	Noradrenaline	27	30	68 [55-79]	66 [55-77]	52	80	100	100	100	100
**Noradrenaline**													

De Backer	Shock, all-causes	First-line in shock	Dopamine	145	135								
Cronin	AMI and CS	CS	Non exposed	95	45					100	100		
Myburgh	Requirement of vasopressor for any cause		Adrenaline	64	64								
Yagi	Cardiovascular shock		Dopamine	98	142								
Tarvasmaki	Acute CS	CS	Non exposed	162	54								
Levy	CS after AMI	CS	Adrenaline	30	27	66 [55-77]	68 [55-79]	80	52	100	100	100	100
**Vasopressin**													
Tarvasmaki	Acute CS	CS	Non exposed	8	208								
**Milrinone**													

Lewis	CS, all etiologies	CS	Dobutamine	50	50	73 [59-81]	75 [67-83]	56	46	4	8		
**Levosimendan**													
Samimi-Fard	STEMI with CS after PCI	CS	Dobutamine	11	11	65±12	63±11	86	75	100	100	100	100
Fuhrmann	Refractory CS <2hrs after PCI	Refractory CS	Enoximone	16	16	68 [60-70]	68 [62-73]	69	56	100	100	100	100
Omerovic	CS due to STEMI	Routine in CS	Non exposed	46	48	65±12	67±11	76	71	100	100	100	100
Huseby	Clinical signs of HF <48hrs after pPCI treated acute STEMI, including CS	Clinical signs of HF	Placebo	4	5					100	100	100	100
Christoph	AMI and CS	CS, refractory	IABP	10	12	68	66	50	67	100	100	100	100

		to noradrenaline +/- dobutamine									
Affronti	CS with VA-ECMO support	Prior to planned weaning ECMO	Non exposed	6	11	57±9	57±8	67	62	50	45
Katsytadze	AMI and CS	CS, also infusion of dopamine	Non exposed	12	15					100	100
Caetano	AMI and CS		Non exposed	12	25	68±11	80±9	83	44	100	100
Tarvasmaki	Acute CS	CS	Non exposed	52	164						

Vally	ICU patients undergoing VA-ECMO		Non exposed	51	99	54±15	53±15	71	63	28	30		
**Dobutamine**													
El Mokhtari	Acute CS	CS, refractory to dopamine alone	Dopexamine	10	10	62±8	66±6	60	70				
Moulopoulos	CS post-MI, therapy prior to IABP initiation	CS, therapy pre-IABP	Non exposed	24	31	62±2/58±5	60±2	92	84	100	100		
Samimi-Fard	STEMI with CS after PCI	CS	Levosimendan	11	11	63±11	65±12	75	86	100	100	100	100
Tarvasmaki	Acute CS	CS	Non exposed	105	111								

Lewis	CS, all etiologies		Milrinone	50	50	75 [67-83]	73 [59-81]	46	56	8	4		
**Dopamine**													
De Backer	Shock, all-causes	First-line in shock	Noradrenaline	135	145								
Yagi	Cardiovascular shock		Noradrenaline	142	98								
Andriange	AMI, subgroup CS	CS	Non exposed	34	11					100	100	0	0
Tarvasmaki	Acute CS	CS	Non exposed	56	160								

Values are presented as mean ± standard deviation or as median [IQR]. Cardiogenic shock patients derived from overall cohort. Revascularization: PCI/CABG. CS= cardiogenic shock; (A)MI= (acute) myocardial infarction; STEMI; ST-segment elevation myocardial infarction; (VA-)ECMO = (venoarterial) extracorporeal membrane oxygenation; PCI= percutaneous coronary intervention; IABP=intraaortic balloon pump; HF=heart failure.

Table S2. Quality assessment of the included randomized trials.

	Random sequence generation	Allocation concealment	Blinding of patients and personnel	Blinding of outcome assessment	Incomplete outcome data	Selective reporting	Other sources of bias	**Risk of Bias**
De Backer	+	+	+	+	+	+		low
Myburgh	+	+	+	?	?	+		Unclear
Samimi-Fard	?	-	-	?	+	-		High
Fuhrmann	+	?	-	-	?	-		High
Huseby	+	+	+	+	+	-		Low
Levy	+	+	+	+	+	+		Low

Table S3. Quality assessment of the included observational studies

	Study population clearly specified and [illegible]	Cohort representative of CS population	Appropriate selection of control group	Therapy and control group selected from same/similar population	Key potential confounding variables measured and adjusted	Therapy and control group same criteria for initiation of treatment	Outcome measure was clearly defined, valid, reliable and implemented	Follow-up long enough for outcomes to occur (> 28 days)	Loss to follow-up after baseline 20% or less	**Total quality score**
Cronin	☆		☆					☆		Low
Yagi	☆		☆							Low
Tarvasmaki	☆	☆					☆	☆	☆	Fair
Andriange	☆	☆		☆		☆	☆	☆		Fair
El Mokhtari	☆			☆		☆	☆	☆		Fair
Moulopoulos	☆		☆			☆		☆		Fair
Omerovic	☆			☆	☆		☆	☆	☆	Fair
Christoph	☆		☆	☆						Low
Affronti	☆			☆		☆	☆			Fair
Katsytadze								☆		Low
Caetano										Low
Lewis	☆		☆	☆			☆		☆	Fair
Vally	☆			☆	☆		☆			Fair

Total quality score grading: low quality = 0-3 stars; fair quality = 4-6 stars, high quality = 7-9 stars

Table S4. Mortality outcomes for cardiogenic shock population.

Study	CS patient population	Control arm	Short-term mortality			Long-term mortality		
			Definition	Therapy	Control	Definition	Therapy	Control
**Adrenaline**								
Myburgh	Acute circulatory failure	Noradrenaline	28-day	15/64 (23)	17/63 (27)	90-day	20/62 (32)	25/61 (41)
Tarvasmaki	CS, 81% ACS	Non exposed				90-day	34/46 (74)	54/170 (32)
Levy	CS after AMI	Noradrenaline	28-day	13/27 (48)	8/30 (27)			
**Noradrenaline**								
De Backer	CS, % AMI ns	Dopamine	28-day	50/145 (34)	64/135 (47)			
Cronin	CS and AMI	Non exposed				ns	79/95 (83)	37/45 (82)
Myburgh	Acute circulatory failure	Adrenaline	28-day	17/63 (27)	15/64 (23)	90-day	25/61 (41)	20/62 (32)
Yagi	Cardiovascular shock, % AMI ns	Dopamine	Acute mortality rates	26/98 (27)	36/142 (25)			
Tarvasmaki	CS, 81% ACS	Non exposed				90-day	76/162 (47)	13/54 (24)
Levy	CS after AMI	Adrenaline	28-day	8/30 (27)	13/27 (48)			

**Vasopressin**								
Tarvasmaki	CS, 81% ACS	Non exposed				90-day	7/8 (88)	81/208 (39)
**Milrinone**								
Lewis	CS, all etiologies (4-8% AMI)	Dobutamine	In-hospital	1/50 (2)	5/50 (10)			
**Levosimendan**								
Samimi-Fard	STEMI, CS after PCI	Dobutamine				1-year	3/11 (27)	1/11 (9)
Fuhrmann	Refractory CS post-MI	Enoximone	30-day	5/16 (31)	11/16 (69)			
Omerovic	CS due to STEMI	Non exposed	30-day	15/46 (33)	17/48 (35)	1-year	20/46 (43)	17/48 (35)
Huseby	CS and clinical signs of HF <48hrs after pPCI treated acute STEMI	Placebo				6-month	1/4 (25)	2/5 (40)
Christoph	AMI and CS	IABP	ns	3/10 (30)	3/12 (25)			
Affronti	VA-ECMO supported CS patients	Non exposed	In-hospital	2/6 (33)	7/11 (64)			
Katsytadze	AMI and CS	Non exposed				1-year	4/12 (33)	8/15 (53)
Caetano	AMI and CS	Non exposed	In-hospital	8/12 (67)	17/25 (68)			

Tarvasmaki	CS, 81% ACS	Non exposed				90-day	17/52 (33)	72/164 (44)
Vally	ICU patients undergoing VA-ECMO	Non exposed	30-day	11/51 (22)	50/99 (51)			
**Dobutamine**								
El Mokhtari	Acute CS, % AMI ns	Dopexamine	24-hrs	0/10 (0)	0/10 (0)	1-year	3/10 (30)	4/10 (40)
Moulopoulos	CS post-MI, therapy prior to IABP initiation	Non exposed	1-month	20/24 (83)	21/31 (68)			
Samimi-Fard	STEMI, CS after PCI	Levosimendan				1-year	1/11 (9)	3/11 (27)
Tarvasmaki	CS, 81% ACS	Non exposed				90-day	50/105 (48)	39/111 (35)
Lewis	CS, all etiologies	Milrinone	In-hospital	5/50 (10)	1/50 (2)			
**Dopamine**								
De Backer	CS, % AMI ns	Noradrenaline	28-day	64/135 (47)	50/145 (34)			
Yagi	Cardiovascular shock, % AMI ns	Noradrenaline	Acute mortality rates	36/142 (25)	26/98 (27)			
Andriange	100% AMI, subgroup CS	Non exposed	30-day	26/34 (76)	11/11 (100)	1-year	27/30 (90)	11/11 (100)

Tarvasmaki	CS, 81% ACS	Non exposed	90-day	24/56 (43)	67/160 (42)

Definitions: mortality= short-term <90 day and long-term ≥ 90-day, (n; number of events/ total at risk (%)); ns=not specified; CS= cardiogenic shock; ACS= acute coronary syndrome; (A)MI= (acute) myocardial infarction; STEMI= ST-segment elevation myocardial infarction; (p)PCI= (primary) percutaneous coronary intervention; HF= heart failure; (VA-)ECMO= (venoarterial) extracorporeal membrane oxygenation; ICU= intensive care unit; IABP= intraaortic balloon pump; HF= heart failure.

Table S5. Primary and secondary outcome(s) of the included studies.

Study	Patient population	Therapy	Primary end-point	Results	Secondary outcome(s)
**Adrenaline**					
Myburgh	Requirement of vasopressor for any cause	Noradrenaline	Achievement of MAP goal > 24hrs	Similar median time to achieving MAP goal and number of vasopressor-free days.	Adrenaline use associated with development tachycardia and lactic acidosis in first 24hrs.
Tarvasmaki	Acute CS	Non exposed	90-day mortality	Adrenaline use associated with excess 90-day mortality.	Adrenaline use associated with increase of cardiovascular and renal biomarkers (i.e. hsTnT, Nt-proBNP and creatinine). Similar CI and BP by 96hrs.
Levy	CS after AMI	Noradrenaline	CI evolution and refractory CS	Similar CI evolution, early termination of study due to higher incidence of refractory CS in adrenaline group.	Similar incidence of arrythmias, higher incidence of refractory CS in adrenaline group.
**Noradrenaline**					
De Backer	Shock, all-causes	Dopamine	28-day mortality	Higher mortality rate in dopamine group (subgroup analysis in CS population; p=0.03).	Dopamine was associated with more arrhythmic events.

Cronin	AMI and CS	Non exposed	Annual incidence of CS over a decade	Annual incidence of CS after AMI decreased, but annual mortality of AMI remained unaltered.	
Myburgh	Requirement of vasopressor for any cause	Adrenaline	Achievement of MAP goal > 24hrs	Similar median time to achieving MAP goal and number of vasopressor-free days.	Adrenaline use associated with development tachycardia and lactic acidosis in first 24hrs.
Yagi	Cardiovascular shock	Dopamine	30-day mortality	Acute mortality rates were significantly lower in dopamine group.	
Tarvasmaki	Acute CS	Non exposed	90-day mortality (adrenaline)		
Levy	CS after AMI	Adrenaline	CI evolution and refractory CS	Similar CI evolution, early termination of study due to higher incidence of refractory CS in adrenaline group.	Similar incidence of arrythmias, higher incidence of refractory CS in adrenaline group.
**Vasopressin**					
Tarvasmaki	Acute CS	Non exposed	90-day mortality (adrenaline)		
**Milrinone**					
Lewis	CS, all etiologies	Dobutamine	Time to resolution of CS	No differences in time to CS resolution, hemodynamic	Similar lenght of ICU stay. More development of arrythmias in

				stabilization (MAP, HR, CI, Sv02) and incidence of inotrope failure.	dobutamine group (63% vs 33%, p<0.01).
**Levosimendan**					
Samimi-Fard	STEMI with CS after PCI	Dobutamine	Cardiac death	No differences in long-term survival.	Greater LVEF after 24 hrs with levosimendan treatment (55 ±8 vs. 45 ±6, p<0.01, no difference at baseline).
Fuhrmann	Refractory CS <2hrs after PCI	Enoximone	30-day mortality	Higher mortality rate in enoximone group vs levosimendan group.	No differences in invasive hemodynamic parameters at 48 hrs, incidence of rhythm disorders or diureses. MOF leading to death occured only in enoximone group (N=4/16), mostly due to acute renal failure.
Omerovic	CS due to STEMI	Non exposed	ns	Similar mortality rates at 30-day and 1-year.	No differences in incidence new-onset AF, in-hospital CA and length of CCU stay.
Huseby	Clinical signs of HF <48hrs after pPCI treated acute STEMI, including CS	Placebo	Change in WMSI (baseline - day 5)	Greater WMSI improvement in levosimendan group.	No differences in changes of NT-proBNP levels, incidence ventricular arrhythmia, AF, infarct size at 6 wk or new clinical event at 6 mth.

Christoph	AMI and CS	IABP	Hemodynamic effects	Similar increase in CI and after 24/48 hrs MAP above target in both groups.	No therapy related complications, no differences in SOFA score at day 1, 2, 3. Three patients died due to MOF in both groups.
Affronti	CS with VA-ECMO support	Non exposed	Weaning outcome ECMO-support	Levosimendan 83% weaning rate vs 27% in non-levosimendan group (p=0.05).	Similar length of ICU stay and number of patients requiring dialysis for acute renal failure.
Katsytadze	AMI and CS	Non exposed	Hemodynamic effects	Levosimendan group higher GFR, ESV and CI at 12 mth, no differences in HR and EF.	
Caetano	AMI and CS	Non exposed	Follow-up regarding MACCE	No difference in MACCE incidence (60.0% vs 50.0%; p = 0.73), namely in re-hospitalization due to heart failure (0% vs 14.3%; p = 0.43).	No differences regarding coronary artery disease severity, incidence of cardio-renal syndrome or in-hospital mortality.
Tarvasmaki	Acute CS	Non exposed	90-day mortality (adrenaline)		
Vally	ICU patients undergoing VA-ECMO	Non exposed	Impact on VA-ECMO weaning	Levosimendan 82% weaning rate vs 62% in non-levosimendan group (p=0.01)	No difference in 30-day mortality after propensity-matching.
**Dobutamine**					

El Mokhtari	Acute CS	Dopexamine	Hemodynamic effects	No difference in hemodynamic effects after 24 hrs (CI, PAP, PCWP, HR, MAP) or myocardial oxygen consumption.	No difference in incidence renal dysfunction or requirement CVVH.
Moulopoulos	CS post-MI, therapy prior to IABP initiation	Non exposed	1-month survival and IABP weaning	None of the 17 patients in the high-dose dobutamine group could be weaned, while 4/7 patients in the low-dose and 10/31 patients in the nondobutamine groups survived.	Difference in urine output between non-dobutamine and high-dose dobutamine group (respectively, 8.0 ±1.3 vs 2.0 ±0.9 mL/hr). No difference in hemodynamic effects (HR, intraarterial SBP, CVP, PWP).
Samimi-Fard	STEMI with CS after PCI	Levosimendan	Cardiac death	No differences in long-term survival.	Greater LVEF after 24 hrs with levosimendan treatment (no difference at baseline).
Tarvasmaki	Acute CS	Non exposed	90-day mortality (adrenaline)		
Lewis	CS, all etiologies	Milrinone	Time to resolution of CS	No differences in time to CS resolution, hemodynamic stabilitzation (MAP, HR, CI, Sv02) and incidence of inotrope failure.	Similar lenght of ICU stay. More development of arrythmias in dobutamine group (63% vs 33%, p<0.01).
**Dopamine**					

De Backer	Shock, all-causes	Noradrenaline	28-day mortality	Higher mortality rate in dopamine group (subgroup analysis in CS population; p=0.03).	Dopamine was associated with more arrhythmic events.
Yagi	Cardiovascular shock	Noradrenaline	30-day mortality	Acute mortality rates were significantly lower in dopamine group.	
Andriange	AMI, subgroup CS	Non exposed	Incidence and prognosis of shock after AMI	Of the 450 cases of AMI, 17.5% (10% CS) developed shock. CS had a worse prognosis compared to other types of shock.	Patients treated with dopamine frequently showed increase in BP and diureses, but also more arrthymias.
Tarvasmaki	Acute CS	Non exposed	90-day mortality (adrenaline)		

Definitions: ns= not specified; CS= cardiogenic shock; MAP= mean arterial pressure; ACS= acute coronary syndrome; CI= cardiac index; (S)BP= (systolic) blood pressure; (A)MI= (acute) myocardial infarction; HR= heart rate; Sv02= mixed venous oxygen saturation; ICU= intensive care unit; STEMI= ST-segment elevation myocardial infarction; (p)PCI= (primary) percutaneous coronary intervention; (LV)E = (left ventricular) ejection fraction; HF= heart failure; MOF= multi-organ failure; AF= atrial fibrillation; CA= cardiac arrest; CCU= coronary care unit; WMSI= wall motion score index; IABP= intraaortic balloon pump; SOFA= sequential organ failure assessment; (VA-)ECMO= (venoarterial) extracorporeal membrane oxygenation; GFR= glomerular filtration rate; ESV= end systolic volume; MACCE= major adverse cardiac and cerebrovascular events; PAP= pulmonary arterial pressure ;P(C)WP= pulmonary (capillary) wedge pressure ;CVVH= continuous venovenous hemofiltration ;CVP= central venous pressure.
